# Mahuang Decoction Antagonizes Acute Liver Failure via Modulating Tricarboxylic Acid Cycle and Amino Acids Metabolism

**DOI:** 10.3389/fphar.2021.599180

**Published:** 2021-03-29

**Authors:** Wenting Liao, Qiwen Jin, Junning Liu, Yiling Ruan, Xinran Li, Yueyue Shen, Zhicheng Zhang, Yong Wang, Shengming Wu, Junying Zhang, Lifeng Kang, Chunyong Wu

**Affiliations:** ^1^ Department of Pharmaceutical Analysis, China Pharmaceutical University, Nanjing, China; ^2^ Institute of Forensic Science, Nanjing Municipal Public Security Bureau, Nanjing, China; ^3^ Nanjing Liuhe District Hospital of Traditional Chinese Medicine, Nanjing, China; ^4^ Department of TCMs Pharmaceuticals, School of Traditional Chinese Pharmacy, China Pharmaceutical University, Nanjing, China; ^5^ Faculty of Medicine and Health, School of Pharmacy, University of Sydney, Sydney, NSW, Australia

**Keywords:** mahuang decoction, acute liver failure, metabolomics, UPLC-Q-exactive-MS, tricarboxylic acid cycle, amino acids metabolism

## Abstract

Acute liver failure (ALF) is a serious clinical disorder with high fatality rates. Mahuang decoction (MHD), a well-known traditional Chinese medicine, has multiple pharmacological effects, such as anti-inflammation, anti-allergy, anti-asthma, and anti-hyperglycemia. In this study, we investigated the protective effect of MHD against ALF. In the lipopolysaccharide and D-galactosamine (LPS/D-GalN)-induced ALF mouse model, the elevated activities of the serum alanine and aspartate transaminases as well as the liver pathological damage were markedly alleviated by MHD. Subsequently, a metabolomics study based on the ultrahigh performance liquid chromatograph coupled with Q Exactive Orbitrap mass spectrometry was carried to clarify the therapeutic mechanisms of MHD against ALF. A total of 36 metabolites contributing to LPS/D-GalN-induced ALF were identified in the serum samples, among which the abnormalities of 27 metabolites were ameliorated by MHD. The analysis of metabolic pathways revealed that the therapeutic effects of MHD are likely due to the modulation of the metabolic disorders of tricarboxylic acid (TCA) cycle, retinol metabolism, tryptophan metabolism, arginine and proline metabolism, nicotinate and nicotinamide metabolism, phenylalanine metabolism, phenylalanine, tyrosine and tryptophan synthesis, as well as cysteine and methionine metabolism. This study demonstrated for the first time that MHD exerted an obvious protective effect against ALF mainly through the regulation of TCA cycle and amino acid metabolism, highlighting the importance of metabolomics to investigate the drug-targeted metabolic pathways.

## Introduction

Acute liver failure (ALF) is a serious clinical disorder that arises from the development of hepatocellular dysfunction, which is predominantly caused by the viral infections (hepatitis A, B and E) in developing world and drug-induced liver injury in developed countries (Bernal and Wendon, 2013; Jiang et al., 2016). To date fatality rates associated with ALF is still as high as 60–80% depending on the disease etiology and patient’s access to care (Patterson et al., 2020). Although the liver transplantation is the best choice for the treatment of ALF, it is clinically limited by many factors, such as lack of available liver organs and immune rejection (Nie et al., 2020). Therefore, it is imperative to seek novel effective medicines for ALF disease.

Mahuang decoction (MHD), a famous prescription in *Treatise on Febrile Disease* (Shang Han Lun in Chinese), consists of *Ephedrae Herba* (Ephedra), *Cinnamomi Ramulus* (Cassia twig), *Armeniacae Semen Amarum* (Bitter apricot kernel) and *Glycyrrhizae Radix* (Prepared licorice), and has been extensively used in treating asthma, cough and cold for thousands of years (He et al., 2018; Huang et al., 2020). Modern research demonstrated that MHD has multiple pharmacological effects, such as anti-inflammation, anti-allergy, anti-asthma, and anti-hyperglycemia (Zheng et al., 2015). It is reported that *Ephedra sinica* Stapf, as well as its two main components pseudoephedrine and ephedrine could prevent lethal liver injury by suppressing hepatocyte apoptosis (Yamada et al., 2008; Wu et al., 2014). As Ephedra is the monarch medicine in MHD and is considered to play a leading role in treating the main syndrome of diseases (He et al., 2018), we hypothesized that MHD is an effective therapeutic strategy for ameliorating ALF.

Evaluating the mechanism of pharmacological action of traditional Chinese medicine (TCM) is difficult because of the unclear active components and their possible synergistic actions (Yang et al., 2015). Metabolomics can comprehensively profile the metabolites in the entire organism that alter in responses to the pathophysiological or drug stimuli, identify their related metabolic pathways, and systematically clarify the mechanism of drug actions (Newgard, 2017; Fu et al., 2019). Thus metabolomics may be a powerful approach to unveil the underlying mechanism of MHD. Recently, ultrahigh performance liquid chromatography (UPLC) coupled with a high resolution mass spectrometer (MS) such as Q-Exactive Orbitrap MS are drawing great attention in metabolomics because of the superior peak resolution, selectivity, sensitivity, reproducibility and analysis speed (Fu et al., 2019; Zhong et al., 2019).

In this study, lipopolysaccharide and D-galactosamine (LPS/D-GalN)-induced ALF mice were used to explore the therapeutic benefits of MHD. The underlying mechanism was clarified by the untargeted metabolomics based on UPLC-Q Exactive Orbitrap MS. Our research uncovered for the first time that MHD could ameliorate ALF mainly through the regulation of TCA cycle and amino acids metabolism, providing new understanding of pathological changes and alternative therapeutic strategies for ALF.

## Materials and Methods

### Chemicals and Reagents

Herbs of Ephedra (*Ephedra sinica* Stapf), Cassia twig (*Cinnamomum cassia* Presl), Bitter apricot kernel (*Prunus armeniaca* L.) and Prepared licorice (*Glycyrrhiza uralensis* Fisch.) were provided by Nanjing Liuhe District Hospital of Traditional Chinese Medicine (Nanjing, China) and were identified by Associate Prof. Junying Zhang from the School of Traditional Chinese Pharmacy of China Pharmaceutical University. Lipopolysaccharide (LPS, from *Escherichia coli*, serotype O55:B5), D-galactosamine (D-GalN), L-2-chlorophenylalanine, amygdalin and trans-cinnamaldehyde were purchased from Aladdin (Shanghai, China). Citric acid, cis-aconitic acid, phenylalanine, tyrosine, tryptophan, valine, arginine, proline, glutamine, pyroglutamic acid, methionine, phenylpyruvic acid, lysine and carnitine were purchased from Shanghai Jingchun Reagent Co., LTD. Lysophosphatidylcholine (LysoPC, 16:0), LysoPC (18:0), and LysoPC (18:2) were obtained from Sigma-Aldrich (St. Louis, MO). Ephedrine hydrochloride, pseudoephedrine hydrochloride and methylephedrine hydrochloride were obtained from National Institutes for Food and Drug Control (Beijing, China). Cinnamic acid was purchased from Nanjing Spring and Autumn Biological Engineering Co., Ltd (Nanjing, China). Glycyrrhizic acid was purchased from Bide Pharmatech Ltd (Shanghai, China). Methanol, acetonitrile, and other reagents were LC/MS grade and obtained from the commercial sources. Water was purified with a Millipore Milli Q-Plus system (Millipore, MA, United States).

### Preparation of MHD

The mixture of Ephedra 3 g, Cassia twig 2 g, Bitter apricot kernel 2 g, and Prepared licorice 1 g was immersed in water for 30 min and extracted twice with 120 ml boiling water for 2 h each time. The extract was filtered and the two filtrates were combined and subsequently concentrated to 40 ml to prepare MHD. HPLC analysis of MHD was performed with Shimadzu LC-20AT system (Shimadzu, Kyoto, Japan) on a Welch Ultimate XB-Phenyl column (4.6 × 250 mm, 5 *μ*m). The column temperature and flow rate was set at 30°C and 1.0 ml/min, respectively. The mobile phase consisted of 0.05% formic acid-0.05% triethylamine aqueous solution (A) and acetonitrile (B) with the gradient elution program as follows: 0–15 min, 8–10% B; 15–20 min, 10% B; 20–50 min, 10–27% B; 50–55 min, 27–38% B; 55–60 min, 38% B; 60–65 min, 38–8% B; 65–75 min, 8% B. The detection wavelengths were 210, 252, 278 and 291 nm (He et al., 2014). The injection volume was 10 *μ*l. The chromatogram of MHD was presented in Supplementary Figure S1. Ephedrine, pseudoephedrine, methylephedrine, amygdalin, cinnamic acid, cinnamaldehyde and glycyrrhizic acid were found in MHD and identified with each standard sample.

### Animals and Treatment

Male ICR mice (25–28 g) were purchased from Comparative Medicine Center of Yangzhou University (Yangzhou, China). All animals were housed in a controlled environment with a 12 h light/dark cycle with *ad libitum* access to food and water. The study was conducted following the protocols approved by the Animal Ethics Committee of China Pharmaceutical University. All animals were randomly divided into three groups including the control group (*n* = 8), model group (*n* = 15) and MHD group (*n* = 8). Mice in the model group were given LPS (10 *μ*g/kg) and D-GalN (700 mg/kg) intraperitoneally to establish an acute liver failure model (Xia et al., 2014). The MHD group was orally administered with MHD (100 mg/kg, calculated as Ephedra) at 12 h and 30 min before exposed to LPS/D-GalN. The control group was treated with equal amounts of saline. Four mice of the model group and one mouse of the MHD group were excluded because of death. At 8 h after treatment of LPS/D-GalN, blood samples were collected from the retro-orbital plexus, clot at room temperature for 1 h, and then centrifuged at 3000 g for 10 min (4 C). The serum was prepared and stored at −80 C immediately until analysis.

### Biochemical Assay

The alanine aminotransferase (ALT) and the aspartate aminotransferase (AST) are important indicators of the liver function (Farooq et al., 2019). ALT and AST in serum were determined using the assay kits purchased from Jiancheng Bioengineering Institute (Nanjing, China) according to the instructions attached to the kits.

### Histopathology

After the mice were sacrificed, the livers were collected and fixed in 4% paraformaldehyde. Then the samples were sent to Pathology and PDX Efficacy Evaluation Center of China Pharmaceutical University for haematoxylin and eosin (H&E) staining.

### Pretreatment of Serum Sample

Prior to analysis, serum samples were thawed on ice and mixed for 5 s at room temperature. An aliquot of 40 μl serum were added to 160 μl pre-cold methanol containing L-2-chlorophenylalanine (2.5 μg/ml) as the internal standard, followed by vortex-mixing for 3 min and then was placed on ice for 20 min. After the mixture was centrifuged at 14000 g for 15 min at 4°C, the supernatant was diluted with 0.1% formic acid at the ratio of 1:1 (v/v) followed by UPLC-Q-Exactive-MS analysis. To verify the repeatability of the LC-MS system, Quality control (QC) samples were prepared by mixing equal aliquots of each sample and were randomly distributed in the real sample sequence (Sangster et al., 2006). In order to avoid the possible signal drift of the mass spectrometer over time, the samples were injected into UPLC-Q-Exactive-MS in a random order.

### UPLC-Q Exactive-MS Analysis

The LC-MS analysis was performed with Q-Exactive Orbitrap coupled to a Ultimate™ 3000 UPLC system (Thermo Fisher Scientific, United States). Analytes were separated on ACQUITY UPLC HSS T3 C18 column (1.8 *μ*m, 2.1 × 100 mm; Waters, Ireland) at 40°C. The mobile phase consisted of 0.1% formic acid solution (A) and acetonitrile containing 0.1% formic acid (B). The gradient program was as following: 5% B at 0–2.5 min, 5–95% B at 2.5–18 min, 95% B at 18–21.5 min, 95–5% B at 21.5–22 min, and 5% B at 22–27 min. The flow rate was 0.4 ml/min. The injection volume was 5 μl. Q Exactive-MS was operated with heated electrospray ionization (HESI)-Ⅱ. Samples were analyzed under positive mode with full-scan and dd MS^2^. The resolution was 70000; mass-to-charge range was from 60 to 900 m/z; sheath gas, aux gas and sweep gas flow rate were set at 40, 10 and 1, respectively; capillary temperature and aux gas heater temperature were set at 320 C and 350 C, respectively; spray voltage was 3.5 kV; S-lens RF level was 50; maximum IT was 200 ms and AGC target was 3e6 at full MS. For dd MS^2^, the resolution was 17500; maximum IT was 50 ms; AGC target was 1e5; normalized collision energy (NCE) was set at 10, 25, and 40; other parameters were same as full-scan mode.

### Data Pre-processing

All the LC-MS raw data were converted to CDF format by Xcalibur 4.2. Data pre-processing, such as peak discrimination, alignment, and matching, were carried out by XCMS package-based R software (Smith et al., 2006). The CentWave algorithm was adopted and algorithm parameters were default settings except for the following: snthresh = 6, peakwidth = c(5, 25), ppm = 30, bw = 5, mzwid = 0.025. The output data matrix contained missing values, which could have a downstream effect on the analysis of the data (Mais et al., 2018). To solve such problem, the variables present in more than 80% of each group were retained (Bijlsma et al., 2006) and the remaining missing values were replaced by a small value that is half of the minimum value in the original data (Mais et al., 2018). After the above steps, the data matrix was normalized by the internal standard.

### Multivariate Analysis

The pre-processed data was imported into SIMCA-P 14.1 (Umetrics, Sweden), followed by multivariate data analysis, such as principle component analysis (PCA), partial least squares discrimination analysis (PLS-DA) and orthogonal partial least squares discrimination analysis (OPLS-DA). The outliers and the general clustering trends were analyzed by PCA. The differences among the control group, model group, and MHD group were observed by PLS-DA. OPLS-DA was utilized to examine the metabolic differences between two groups. Besides, all PLS-DA and OPLS-DA models were subjected to the permutation test.

### Metabolites Identification and Metabolic Pathway Analysis

Metabolite identification was performed according to our previous report (Tan et al., 2018). Briefly, the quasi-molecular ions were judged according to the positive scanning in MS. The molecular information was obtained from a freely accessible database of HMDB (Wishart et al., 2018), METLIN (Guijas et al., 2018) and KEGG (Kanehisa et al., 2014) within a mass accuracy of 10 ppm. To narrow the scope of target metabolites, the quasi-molecular ions were then subjected to MS/MS analysis. The affected metabolic pathways were analyzed and visualized via MetaboAnalyst (https://www.metaboanalyst.ca/) with identified differential metabolites (Chong et al., 2018, 2019). Finally, 17 available standards were adopted to confirm the identified metabolites.

### Statistical Analysis

The normality and homogeneity of variance of all samples were tested by IBM SPSS Statistics 26. One-way ANOVA or Kruskal-Wallis was used to test the statistical differences of the samples according to whether they obey normal distribution and homogeneity of variance. The resultant *p* values were corrected by Bonferroni correction to *∝*/*n*, where *∝* = 0.05 and *n* is the number of comparisons in statistical analysis (Berben et al., 2012; Araújo et al., 2018). Adjusted *p* < 0.05 was considered statistically significant.

## Results

### Biochemical Assay and Pathological Changes

**FIGURE 1:**
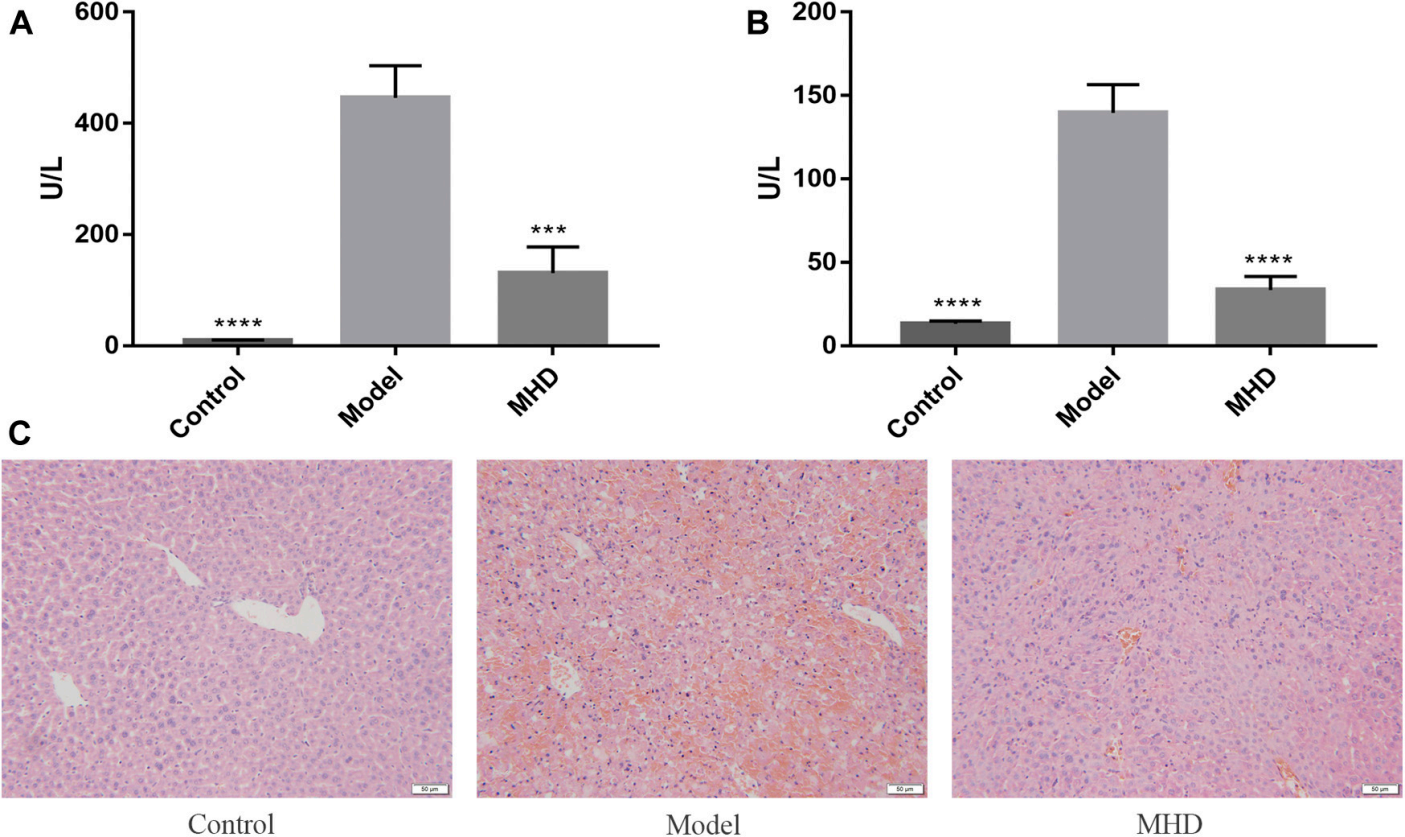
Effects of MHD on serum ALT **(A)** and AST **(B)** levels in mice with acute liver failure induced by LPS/D-GalN. Values ​​are shown in Mean ± SEM. ***p < 0.001, ****p < 0.0001 vs model group. **(C)** Images of H&E stained liver cells (original magnification ×200).

The serum ALT and AST levels of each group are shown in Figures 1A,B, where ALT reflects the degree of liver cell damage and AST reflects the degree of hepatocyte necrosis. The levels of ALT together with AST in the model group were significantly higher than those in the control group, and were similar to the levels reported previously (Wu et al., 2014). Meanwhile, the notably decreased levels of ALT and AST were observed in MHD groups. Consistent with this result, large areas of hepatocytes necrosis were visible in model group by H&E staining, which was remarkably improved by the treatment of MHD (Figure 1C), demonstrating that MHD has a hepatoprotective effect.

### Metabolic Profiling and Multivariate Analysis

The typical total ion current (TIC) chromatogram of UPLC-Q-Exactive MS is shown in Supplementary Figure S2. The relative standard deviation (RSD) for the peak intensity of the internal standard was less than 3.0% in QC samples. After peak alignment, filtering and normalization, the RSD of intensity of all peaks in QC samples was calculated. The RSD of 30% covered 88.0% features, indicating that the analytical method had good repeatability. With the RSD less than 30% in QC samples, 7770 molecular features were obtained for further multivariate data analysis.

**FIGURE 2:**
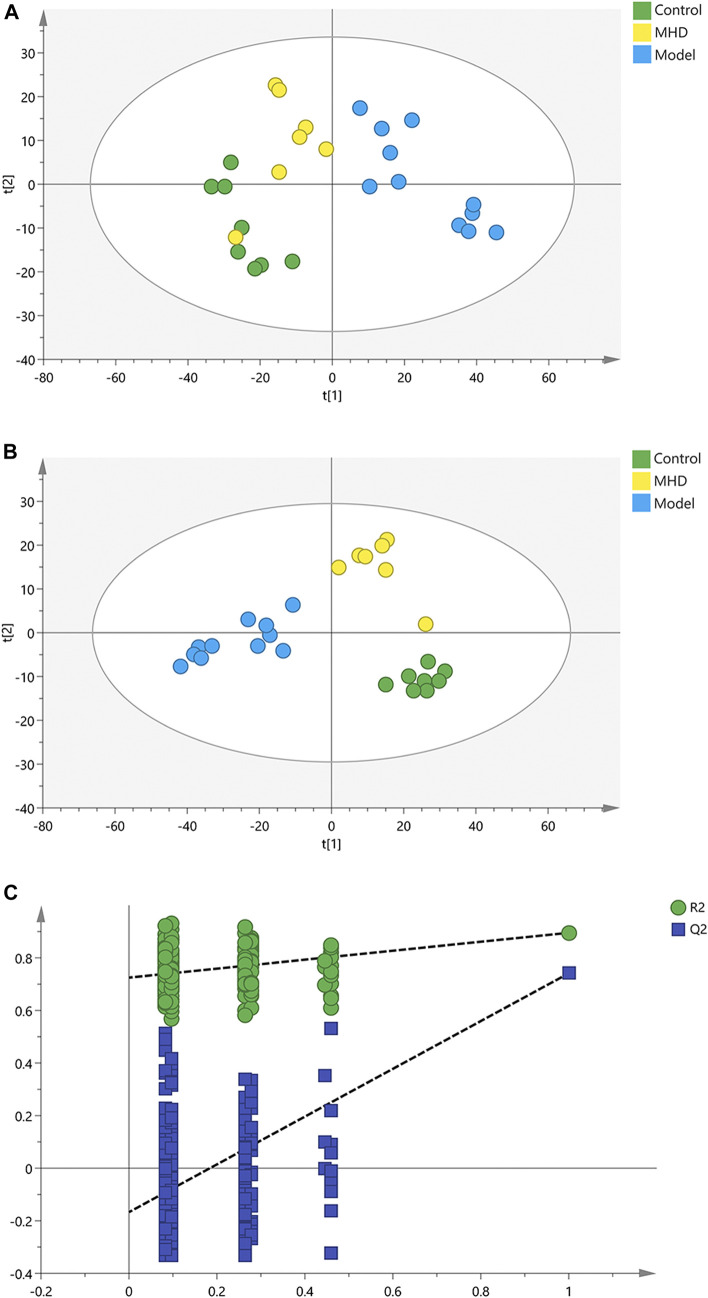
PCA, PLS-DA and permutation test of serum samples. **(A)** PCA score scatter plots. **(B)** PLS-DA score scatter plots. **(C)** Permutation tests of PLS-DA.

To analyze the protective effect of MHD, PCA and PLS-DA models were established. An unsupervised PCA model was carried out to observe the tendency of MHD group separated from model group and control group. As shown in Figure 2A, a separated trend of the inter-group was observed on the scores plot. The main parameter of PCA, namely R^2^X value, was greater than 0.4, indicating that the models were well-fitted. As one of the supervised analysis, PLS-DA could ignore intra-group errors as well as random errors, and focus on the analysis of differences between groups. PLS-DA scores plot showed good discrimination power among control, model and MHD groups (Figure 2B). The predictive capability of the model was assessed by the internal validation (R^2^Y = 0.937, Q^2^ = 0.824), suggesting the goodness of fit and predictive capability of the model. A random permutation test (200 times) was further used to validate the reliability of the PLS-DA model. As R^2^cum and Q^2^cum values were lower than the original values of the validation plot (Figure 2C), the results were non-overfitting and reliable.

### Identification of Differential Metabolites

**FIGURE 3:**
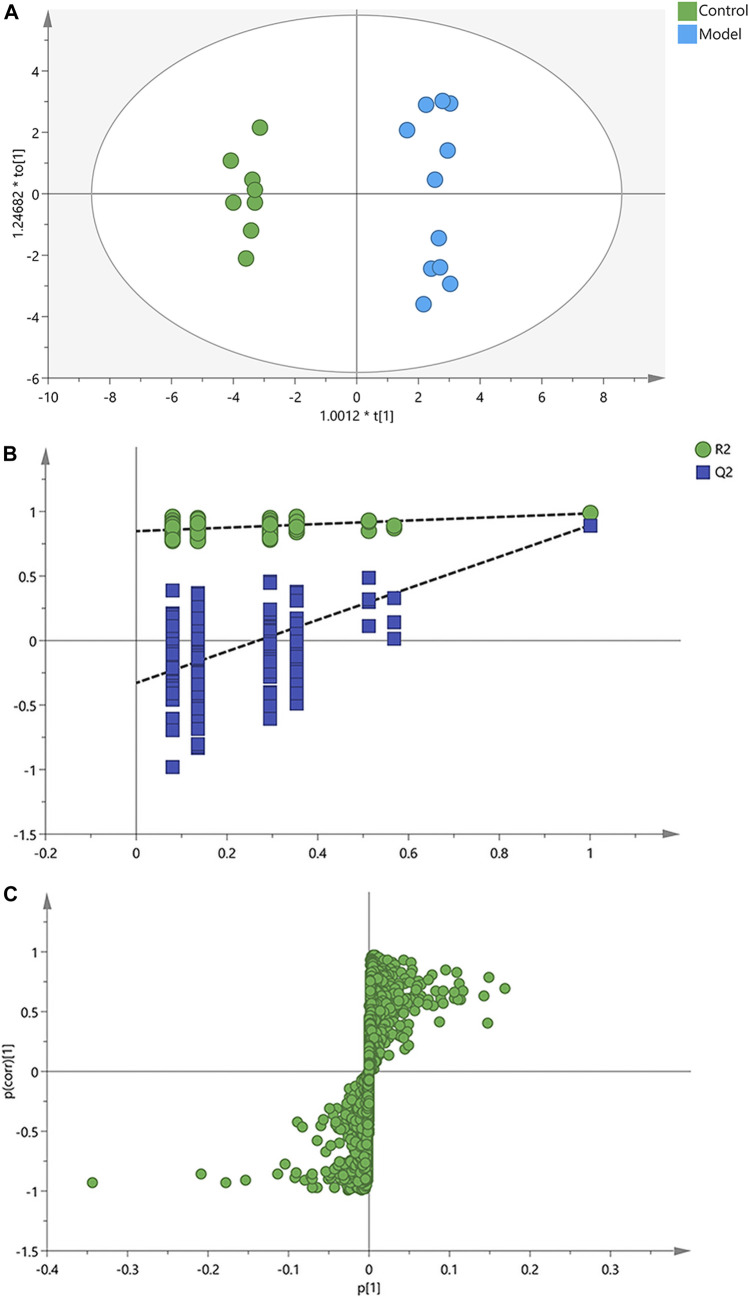
OPLS-DA score plots, permutation test and S-plots of serum samples. **(A)** OPLS-DA score plots. **(B)** Permutation tests of OPLS-DA. **(C)** S-plots obtained from OPLS-DA model.

Afterwards, OPLS-DA was applied to search the differential metabolites between the control and model groups, and a good discrimination between groups was observed (Figure 3A). The predictive capability of the model was assessed by the internal validation (R^2^Y = 0.985, Q^2^ = 0.893), suggesting a satisfactory fit with high predictive power. In the random permutation test (200 times), R^2^cum and Q^2^cum values in permuted classes were lower than those in original classes, revealing that the OPLS-DA models were not over-fitting (Figure 3B). The S-plot generated from OPLS-DA model was used to find potential biomarkers, since the variables distributed at both ends of the S-plot markedly contribute to the clustering and discrimination (Figure 3C).

**FIGURE 4:**
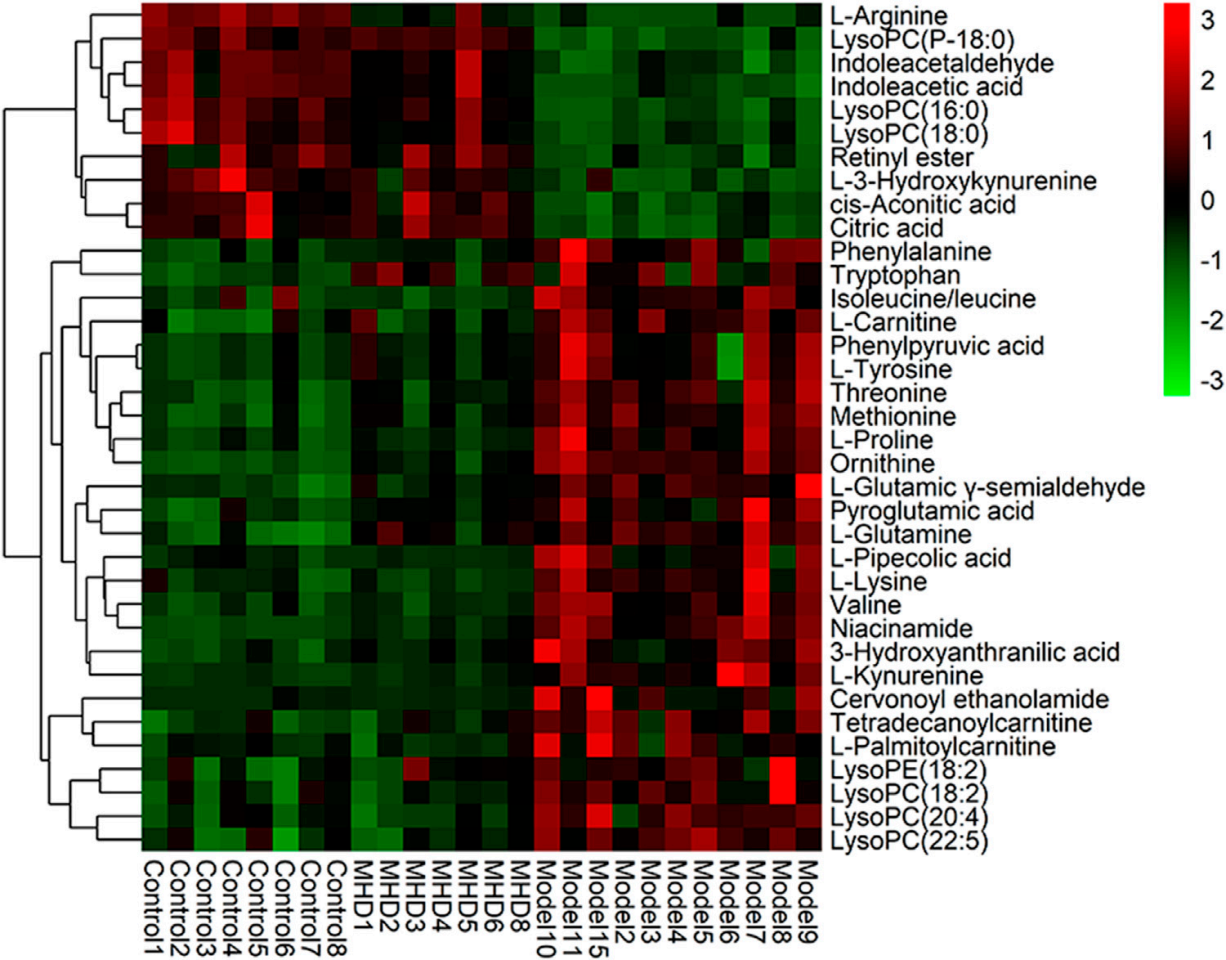
Heatmap of the protective activity of MHD for ALF. The degree of change is marked with different colors. Red and green indicate up-regulation and down-regulation respectively. Each row represents a metabolite. Each column represents an individual sample.

**FIGURE 5:**
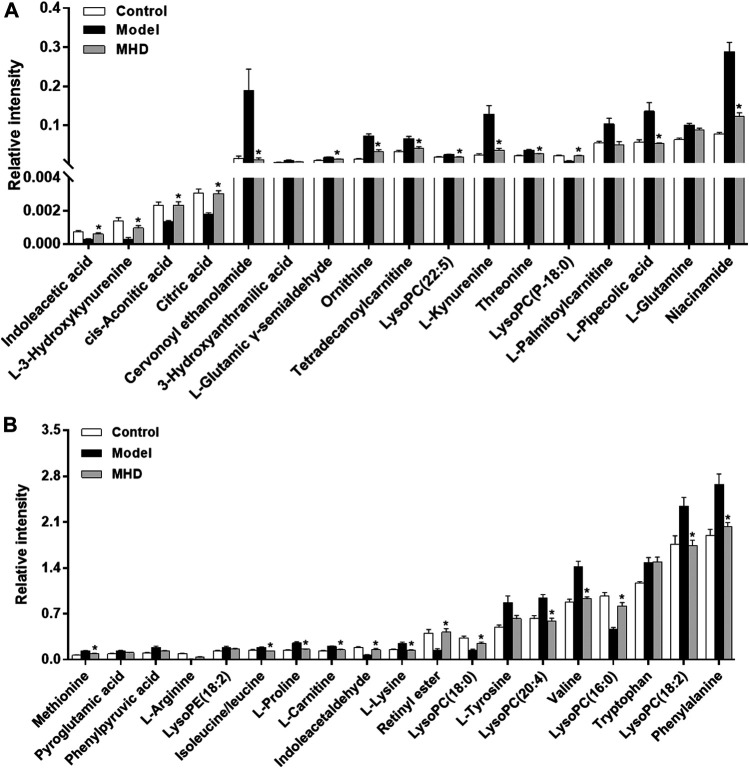
Changes in the relative intensity of target metabolites. Statistical significance was performed using univariate statistical analysis. 27 metabolites were significantly reversed by MHD. Values ​​are shown in Mean ± SEM. *p < 0.05 vs. model group.

Differential metabolites between the model and control groups were identified by using a threshold of variable importance in the projection (VIP) values (VIP>1.0) produced from the OPLS-DA model (Fan et al., 2016). After restricting with univariate statistical analysis (*p* < 0.05), 36 significantly changed metabolites were designated as biomarkers of ALF (Table 1). The MS/MS spectra of these biomarkers together with their proposed fragmentation pathways are presented in Supplementary Figure S3. The relative levels of these biomarkers in each mouse were visualized in Figure 4. Compared to the model group, the relative intensity of most biomarkers were reverted by MHD, among which 27 metabolites were significantly regulated (Figure 5).

**Table 1:** Changes of metabolites in mice with acute liver failure.

Metabolites		Experimental [M + H]^+^ (*m/z*)			Retention times (min)			Adduction			Theoretical [M + H]^+^ (*m/z*)			MS fragments			Delta (ppm)			Trend			Fold Change					
																							CON/M			MHD/M			
L-Proline^a^		116.0707			0.673			M + H			116.0706			70.07			1			↓			0.6**			0.6*			
Valine^a^		118.0863			0.684			M + H			118.0863			72.08, 55.06			0			↓			0.6**			0.7**			
Threonine^b^		120.0655			0.653			M + H			120.0655			102.06, 74.06, 60.99			0			↓			0.6****			0.8**			
Niacinamide^b^		123.0553			1.000			M + H			123.0553			106.03, 79.02			0			↓			0.3****			0.5***			
Pyroglutamic acid^a^		130.0498			0.652			M + H			130.0499			84.04, 70.07, 56.06			1			↓			0.7**			0.8			
L-Pipecolic acid^b^		130.0862			0.633			M + H			130.0863			112.04, 84.04			1			↓			0.4*			0.4*			
L-Glutamic *?*-semialdehyde^b^		132.0656			0.653			M + H			132.0655			114.07, 86.06			1			↓			0.6***			0.8*			
Isoleucine/leucine^b^		132.1018			0.765			M + H			132.1019			86.06, 56.97			1			↓			0.8**			0.7*			
Ornithine^b^		133.0970			0.553			M + H			133.0972			116.07, 70.07			1			↓			0.2****			0.5*			
L-Glutamine^a^		147.0762			0.648			M + H			147.0764			130.05, 102.06, 84.04			1			↓			0.6**			0.9			
L-Lysine^a^		147.1126			0.558			M + H			147.1128			130.09, 84.08, 56.05			1			↓			0.6**			0.6**			
Methionine^a^		150.0581			0.710			M + H			150.0583			133.03, 104.05			1			↓			0.5***			0.7*			
3-Hydroxyanthranilic acid^b^		154.0496			1.262			M + H			154.0499			136.04, 109.96, 67.05			2			↓			0.5**			0.7			
Indoleacetaldehyde^b^		160.0755			1.576			M + H			160.0757			141.92, 131.97			1			↑			2.7***			2.0*			
L-Carnitine^a^		162.1122			0.647			M + H			162.1125			103.04, 85.03, 60.08			2			↓			0.7***			0.8**			
Phenylpyruvic acid^a^		165.0544			1.070			M + H			165.0546			147.04, 119.05			1			↓			0.6**			0.7			
Phenylalanine^a^		166.0860			2.057			M + H			166.0863			149.06, 131.05, 120.08			2			↓			0.7**			0.8*			
cis-Aconitic acid^a^		175.0234			0.996			M + H			175.0237			157.11, 61.53			2			↑			1.7**			1.7**			
L-Arginine^a^		175.1187			0.595			M + H			175.1190			158.00, 70.07, 60.06			2			↑			10.7****			3.7			
Indoleacetic acid^a^		176.0704			1.577			M + H			176.0706			158.00, 106.99			1			↑			2.6***			2.0*			
L-Tyrosine^a^		182.0809			1.070			M + H			182.0812			165.05, 136.08			2			↓			0.6**			0.7			
Citric acid^a^		193.0344			0.996			M + H			193.0343			175.03, 133.01, 61.04			1			↑			1.7****			1.6****			
Tryptophan^a^		205.0968			4.374			M + H			205.0972			188.07, 170.06			2			↓			0.8*			1.0			
L-Kynurenine^b^		209.0917			1.972			M + H			209.0921			192.07, 120.04, 94.07			2			↓			0.2***			0.4*			
L-3-Hydroxykynurenine^b^		225.0865			1.049			M + H			225.0870			207.02, 179.02, 161.01			2			↑			5.1**			3.1**			
Retinyl ester^b^		303.2311			15.346			M + H			303.2319			285.22, 267.21, 256.88			3			↑			2.8***			2.7***			
Tetradecanoylcarnitine^b^		372.3099			13.325			M + H			372.3108			313.24, 85.03, 60.08			2			↓			0.5**			0.7*			
Cervonoyl ethanolamide^b^		373.2728			11.073			M + H			373.2737			355.26, 241.19, 161.13, 81.07			2			↓			0.1**			0.1**			
L-Palmitoylcarnitine^b^		400.3412			14.522			M + H			400.3421			341.26, 144.10, 85.03			2			↓			0.5*			0.6			
LysoPE(18:2)^b^		478.2918			14.002			M + H			478.2928			460.28, 337.27			2			↓			0.7*			0.8			
LysoPC(16:0)^a^		496.3387			14.260			M + H			496.3398			258.11, 184.07			2			↑			2.1****			1.7*			
LysoPC(P-18:0)^b^		508.3751			15.304			M + H			508.3762			184.07, 125.00, 104.11, 86.10			2			↑			2.5****			2.3****			
LysoPC(18:2)^a^		520.3387			14.063			M + H			520.3398			184.07, 104.11			2			↓			0.8**			0.8**			
LysoPC(18:0)^a^		524.3699			15.761			M + H			524.3711			506.36, 184.07, 86.10			2			↑			2.2**			1.6*			
LysoPC(20:4)^b^		544.3386			14.115			M + H			544.3398			184.07, 104.11, 86.10, 60.08			2			↓			0.7**			0.7**			
LysoPC(22:5)^b^		570.3540			14.459			M + H			570.3554			387.29, 184.07,104.11			2			↓			0.7*			0.8*			

Delta = (abs (experimental *m/z*–theoretical *m/z*)/theoretical *m/z*) × 1000000. Trend: change trend of contents of metabolites in each group compared to the model group. Fold change: relative amount of each group compared to the model group. *adjusted *p* < 0.05, **adjusted *p* < 0.01, ***adjusted *p* < 0.001, ****adjusted *p* < 0.0001, adjusted *p*: *p* values corrected by Bonferroni.

^a^ Metabolites validated with standard sample.

^b^ Metabolites putatively annotated. Abbreviations: LysoPE, lysophosphatidyl ethanolamine; LysoPC, Lysophosphatidylcholine.

### Metabolic Pathways Analysis

**FIGURE 6:**
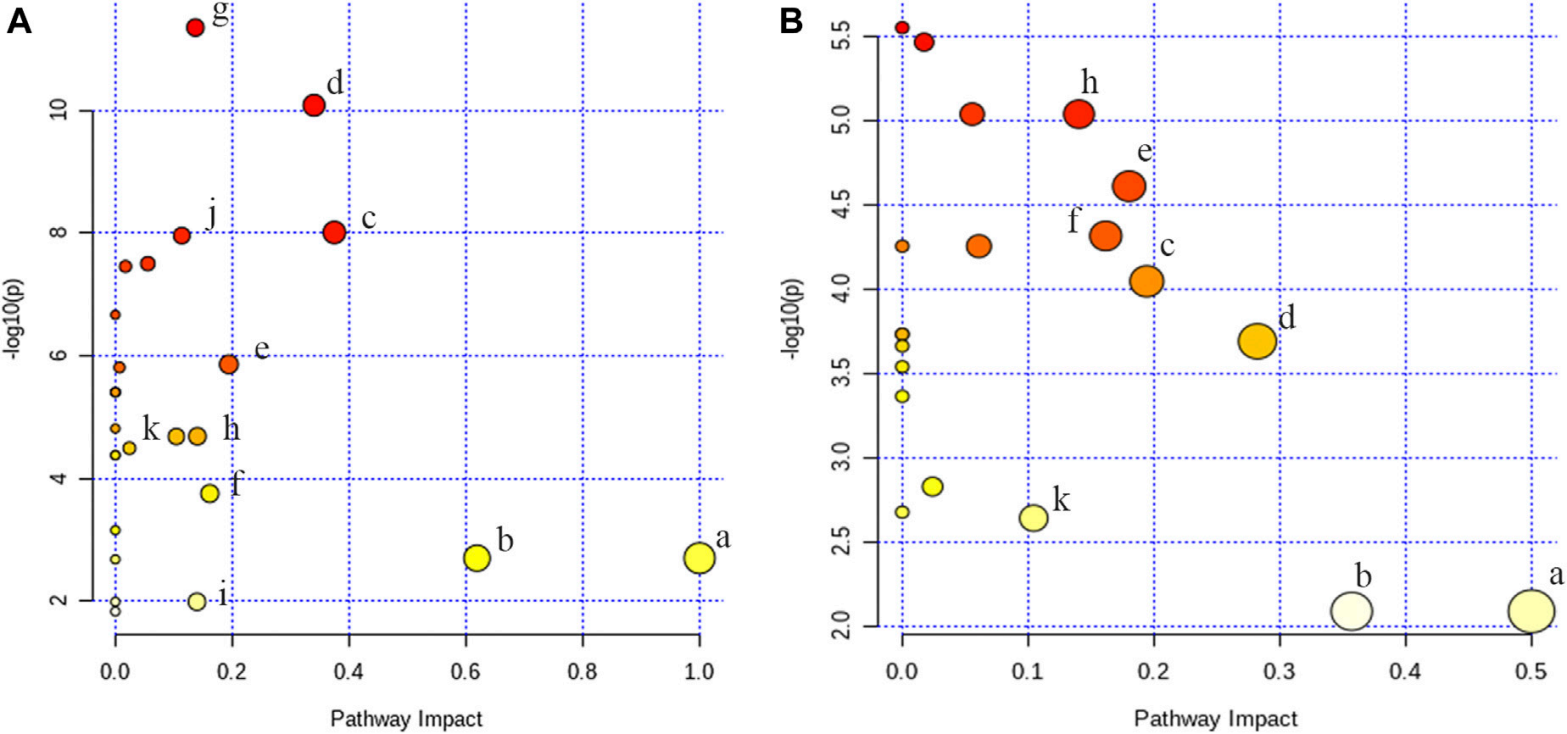
Analysis of metabolic pathways **(A)** control vs model **(B)** MHD vs model. a: phenylalanine, tyrosine and tryptophan biosynthesis, b: phenylalanine metabolism, c: tryptophan metabolism, d: arginine and proline metabolism, e: nicotinate and nicotinamide metabolism, f: retinol metabolism, g: arginine biosynthesis, h: TCA cycle, i: tyrosine metabolism, j: alanine, aspartate and glutamate metabolism k: cysteine and methionine metabolism.

**FIGURE 7:**
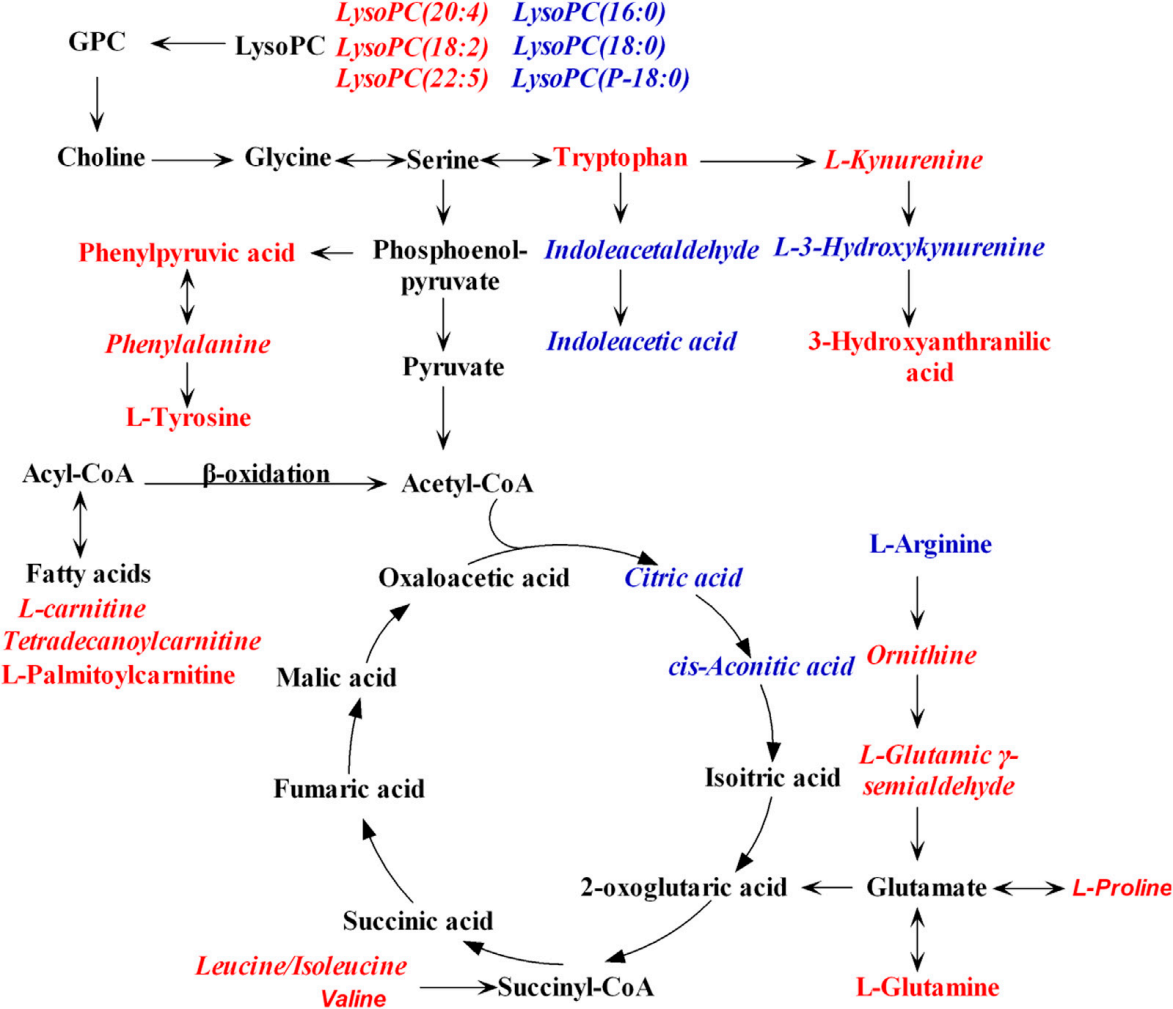
Schematic metabolic network of LPS/D-GalN-induced ALF and MHD modulation based on the KEGG database. Red: the levels of metabolites in model group were up-regulated compared to control group. Blue: the levels of metabolites in model group were down-regulated compared to control group. The italic metabolite names were significantly reversed by MHD treatment. Abbreviations: LysoPC, Lysophosphatidylcholine; GPC, Glycerophosphocholine.

To figure out the potential LPS/D-GalN-induced and MHD-modulated metabolic pathways, 36 significantly altered metabolites in ALF model group (vs. control group) and 27 significantly reverted metabolites in MHD group (vs. model group) were imported into MetaboAnalyst 4.0. By using a threshold of the impact-value (≥0.10), 11 metabolic pathways were identified to be the potential targets of ALF, including phenylalanine, tyrosine and tryptophan biosynthesis, phenylalanine metabolism, tryptophan metabolism, arginine and proline metabolism, nicotinate and nicotinamide metabolism, retinol metabolism, arginine biosynthesis, tricarboxylic acid (TCA) cycle (citric acid cycle), tyrosine metabolism, alanine, aspartate and glutamate metabolism, as well as cysteine and methionine metabolism (Figure 6A and Supplementary Table S1). MHD-regulated metabolic pathways were identified to be TCA cycle, retinol metabolism, tryptophan metabolism, arginine and proline metabolism, nicotinate and nicotinamide metabolism, phenylalanine metabolism, phenylalanine, tyrosine and tryptophan synthesis, as well as cysteine and methionine metabolism (Figure 6B and Supplementary Table S2). A schematic metabolic network of LPS/D-GalN-induced ALF and MHD modulation were analyzed with the KEGG database (Kanehisa et al., 2014) by relating the major metabolic pathways (Figure 7 **)**.

## Discussion

Mahuang decoction (MHD) has been widely utilized to treat asthma, cough, and exogenous wind-cold (He et al., 2018; Huang et al., 2020). The monarch medicine of MHD, *Ephedra sinica* Stapf, and its two main components pseudoephedrine and ephedrine have been reported to prevent lethal liver injury (Yamada et al., 2008; Wu et al., 2014). Therefore, we hypothesized that MHD has protective effect against ALF.

To verify our hypothesis, the serum transaminase test and histopathology together with a UPLC-Q-Exactive-MS-based metabolomics study were performed to explore the potential efficacy and mechanisms of MHD against ALF. The serum ALT and AST levels in LPS/D-GalN-induced ALF mice were markedly reduced with the treatment of MHD, providing strong evidences for the protective effect of MHD on ALF. Furthermore, a total of 36 metabolites were identified to be ALF relevant biomarkers, among which 27 metabolites were significantly reverted by the treatment of MHD. By constructing the relevant metabolic pathways of these reverted biomarkers, eight metabolic pathways were filtered out to be the most important pathways for the anti-ALF efficacy of MHD, including TCA cycle, retinol metabolism, tryptophan metabolism, arginine and proline metabolism, nicotinate and nicotinamide metabolism, phenylalanine metabolism, phenylalanine, tyrosine and tryptophan synthesis, as well as cysteine and methionine metabolism.

### TCA Cycle and Energy Metabolism

The TCA cycle is essential for the aerobic metabolism, which could facilitate the adequate throughput of the substrates derived from carbohydrates, fatty acids and certain amino acids. The two pivotal intermediates, namely citric acid and cis-aconitic acid, were altered following LPS/D-GalN inducement, suggesting that ALF could perturb TCA cycle. This result is supported by the previous studies (Carvalho et al., 2002; Dabos et al., 2011; Pathania et al., 2020). An unbalanced TCA cycle was indicative of the decreased oxidative metabolism. After treating with MHD, the levels of citric acid and cis-aconitic acid were higher than that in ALF mice, demonstrating that TCA metabolism tended to be normal.

As the principal precursors of *ß*-oxidation substrates and acylcarnitines in serum, the long-chain acylcarnitines (LCAC) mainly come from the liver (Sandor et al., 1990; Jones et al., 2010). The levels of LCAC including tetradecanoylcarnitine and palmitoylcarnitine were significantly increased in ALF group compared to the control group, implying that ALF condition could inhibit the fatty acid oxidation. This finding is supported by the previous studies in which acylcarnitines were elevated under ALF condition (Bi et al., 2013; McGill et al., 2014). The high levels of LCAC in ALF mice may be related to the downregulation of carnitine palmitoyltransferase I (Cpt1) and acyl-CoA thioesterase 1 (Acot1) genes that are involved in the fatty acid *ß*-oxidation pathway (Bi et al., 2013). After the treatment of MHD, the level of tetradecanoylcarnitine was significantly reverted, demonstrating that the modulation of herapeutic fatty acid *ß*-oxidationt is involved in the pharmacological process of MHD.

Branched chain amino acids (BCAAs) are important energy substrates, including valine, leucine, and isoleucine, and they enter the TCA cycle through deamination, decarboxylation, and *ß*-oxidation processes. The serum levels of BCAAs were up-regulated in ALF mice, while down-regulated towards normal when treated with MHD. A possible mechanism is that the TCA cycle in mice is inhibited under the condition of ALF, leading to a reduction of ATP production. Energy compensation may be achieved by consuming BCAAs and accelerating *ß*-oxidation of fatty acids since the level of citric acid and cis-aconitic acid in TCA cycle were elevated but the level of BCAAs and LCAC were reduced after MHD treatment.

Together, these results suggest that the hepatoprotective activities of MHD against ALF are highly likely due to the modulation of the energy metabolism in liver.

### Amino Acids Metabolism

The serum levels of aromatic amino acids (AAA) including phenylalanine, tyrosine and tryptophan were elevated in ALF mice. A previous study has implied that the levels of AAA, especially phenylalanine and tryptophan, are good biomarkers of ALF severity, which is probably caused by the inefficient degradation or gluconeogenesis-based conversion of AAA in the damaged liver (Cao et al., 2012). Beside, elevated AAA is closely related with hepatic encephalopathy associated with ALF (Dejong et al., 2007; Wijdicks, 2016). After the treatment of MHD, the serum level of phenylalanine was significantly reverted, suggesting that MHD can promote the recovery of liver function and thus reduce the risk of hepatic encephalopathy.

Consistent with previous reports (Clària et al., 2019; Sekine and Fukuwatari, 2019), the present study observed that tryptophan metabolism is one of the significantly disturbed pathways in ALF mice. This abnormality was regulated by MHD treatment, and similar activity was observed in other TCM such as Yin-Chen-Hao Tang (Liu et al., 2019). Tryptophan and its metabolites play critical roles in many physiological processes, such as inflammation, immune response, and neurotransmission (Cervenka et al., 2017). Free tryptophan is mainly converted to kynurenine with the involvement of microglial indoleamine-2,3-dioxygenase 1, and the process could be stimulated by inflammation (Platten et al., 2019). The substrates of kynurenine pathways increased by kynurenine metabolism will transfer to the central nervous system (CNS) and be degraded locally, which eventually damage the CNS (Müller and Schwarz, 2007). Compared with the control group, the serum level of kynurenine is up-regulated in model group but reverted by MHD, which indicates that MHD may have anti-inflammatory effect.

Moreover, arginine and proline metabolism was disturbed in ALF mice while reverted by MHD. A similar activity was observed in Yin-Chen-Hao Tang (Liu et al., 2019). L-Arginine is an essential amino acid. Catabolic diseases such as cancer and injury will increase the utilization of arginine, leading to arginine consumption. In ALF mice, arginine levels were significantly decreased (>10-fold change, adjusted *p* < 0.001) and the levels of intermediates of arginine metabolism such as ornithine and L-glutamic-*γ*-semialdehyde were increased, suggesting that ALF caused excessive consumption of arginine. With the treatment of MHD, the level of ornithine and L-glutamic-*γ*-semialdehyde were down-regulated, indicating the disturbance of arginine metabolism was alleviated to a certain extent.

## Conclusion

In this study, the hepatoprotective activity of MHD against ALF was confirmed by the serum transaminase test and histopathology, and the underlying mechanisms was studied by the UPLC-Q Exactive-MS-based metabolomics. We have identified 36 biomarkers of ALF, among which the abnormalities of 27 metabolites were regulated by the treatment of MHD. Metabolic pathway analysis revealed that the anti-ALF mechanisms of MHD may be mainly attributed to the modulation of metabolic disorders of TCA cycle and amino acids metabolism. To obtain a more complete understanding of MHD, future studies is necessary to find the key factors of the identified metabolic pathways and determine their relationship with the chemical components of MHD.

## Data Availability

The raw data supporting the conclusions of this article will be made available by the authors, without undue reservation, to any qualified researcher.
