# Supplementary material for: Mahuang Decoction Antagonizes Acute Liver Failure via Modulating Tricarboxylic Acid Cycle and Amino Acids Metabolism
Source: Front Pharmacol. 2021 Mar 29;12:599180. doi: 10.3389/fphar.2021.599180 (PMC8043081; doi:10.3389/fphar.2021.599180)
Supplement: Supplementary file 1 [file DataSheet1.docx]

**Supplementary information**

**Contents of supporting information**

**Figure S1**. The HPLC chromatogram of MHD. 1. ephedrine, 2. pseudoephedrine, 3. methylephedrine, 4. amygdalin, 5. cinnamic acid, 6. cinnamaldehyde, 7.glycyrrhizic acid**.**

**Figure S2.** The typical total ion current (TIC) chromatogram of UPLC-Q Exactive-MS.

**Figure S3.** The typical MS/MS spectra and proposed fragmentation pathway of identified metabolites.

**Table SI**. Analyzed pathways of metabolomics data differently regulated in serum of ALF model mice.

**Table S2**. Analyzed pathways of metabolomics data differently regulated in serum of MHD mice compared with model mice.

**
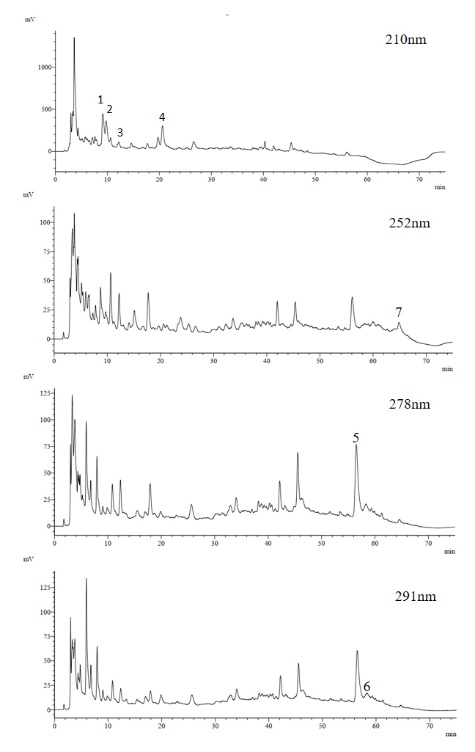
**

**Figure S1**. The HPLC chromatogram of MHD. 1. ephedrine, 2. pseudoephedrine, 3. methylephedrine, 4. amygdalin, 5. cinnamic acid, 6. cinnamaldehyde, 7.glycyrrhizic acid**.**

**Figure S2.** The typical total ion current (TIC) chromatogram of UPLC-Q Exactive-MS.


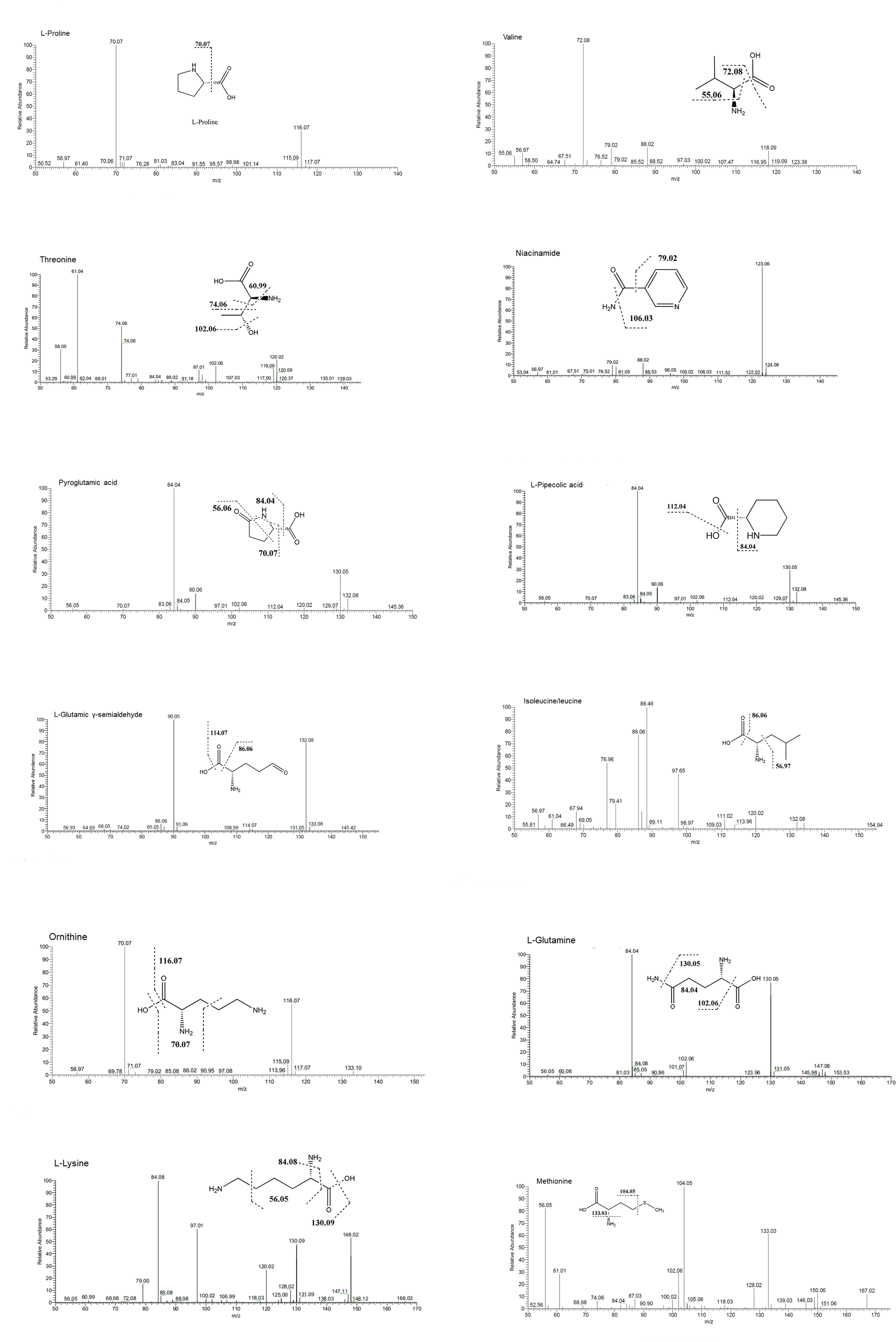


**Figure S3.** The typical MS/MS spectra and proposed fragmentation pathway of identified metabolites.


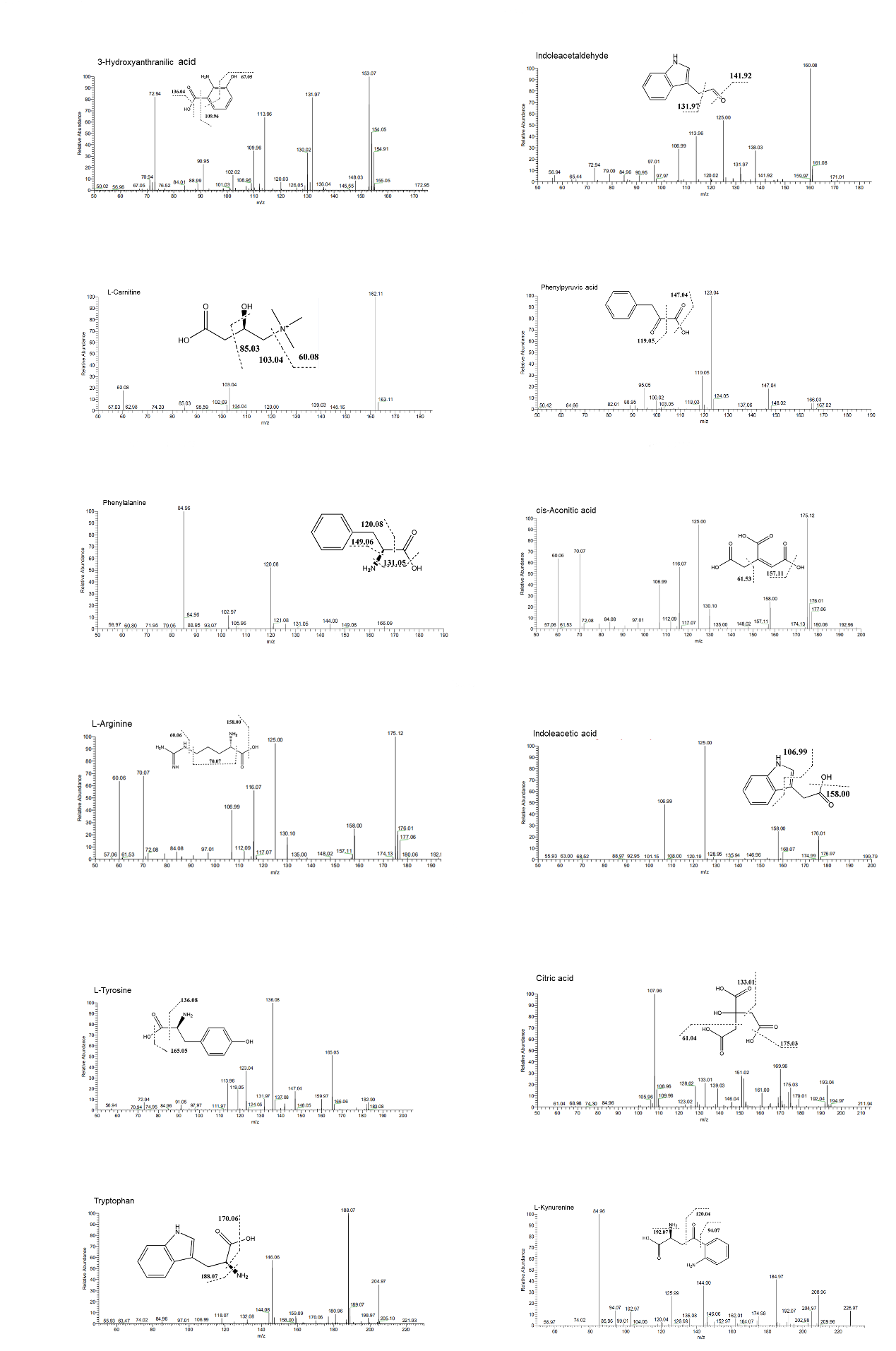


**Figure S3.** The typical MS/MS spectra and proposed fragmentation pathway of identified metabolites (continued).


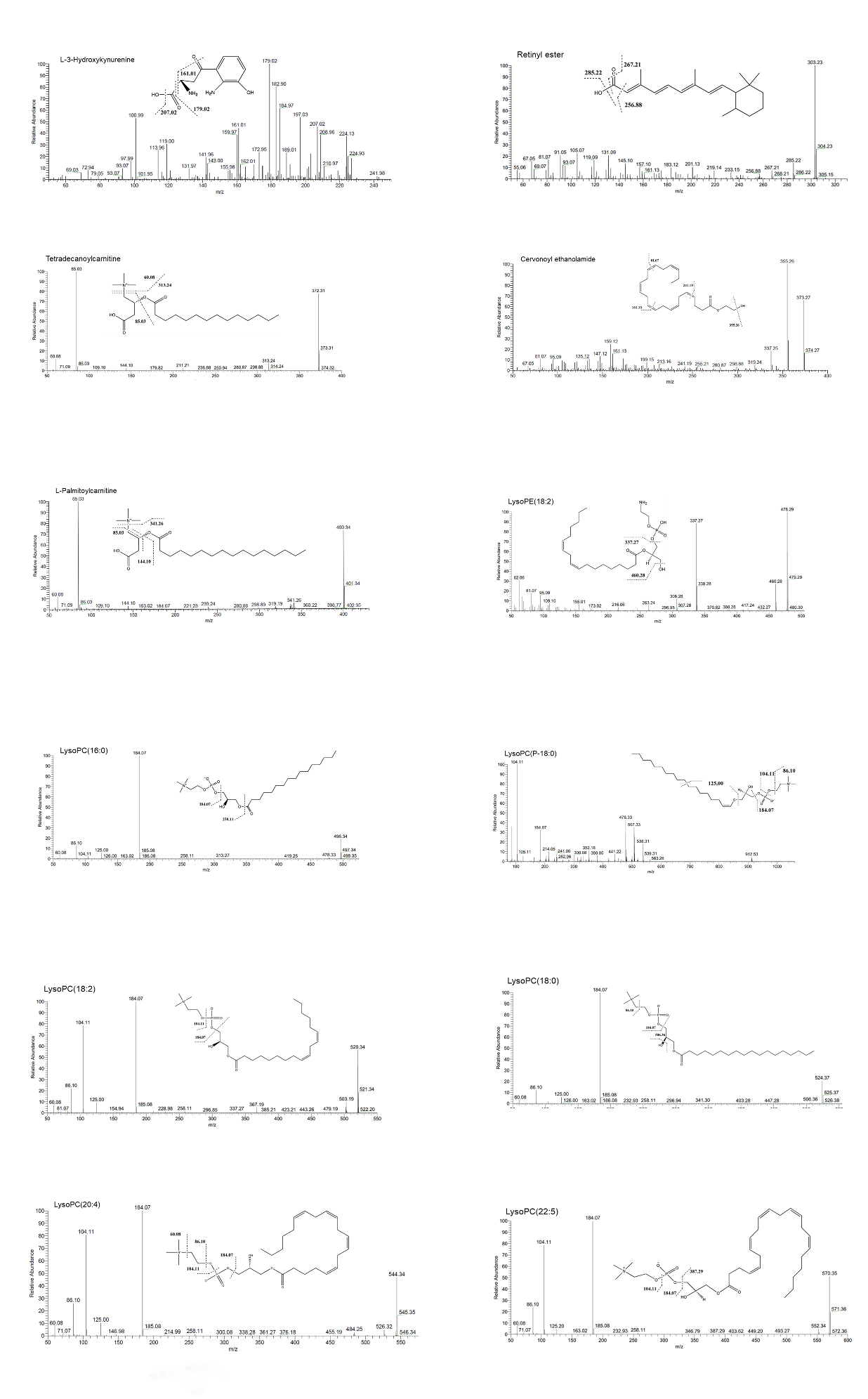


**Figure S3.** The typical MS/MS spectra and proposed fragmentation pathway of identified metabolites (continued).

| **Table S1.** Analyzed pathways of metabolomics data differently regulated in serum of ALF model mice. | | | | | | |
| --- | --- | --- | --- | --- | --- | --- |
|  | Total Cmpd | Hits | Raw p | -log(p) | FDR | Impact |
| Phenylalanine, tyrosine and tryptophan biosynthesis | 4 | 3 | 2.00E-03 | 2.70 | 2.34E-03 | 1.00 |
| Phenylalanine metabolism | 12 | 3 | 2.00E-03 | 2.70 | 2.34E-03 | 0.62 |
| Tryptophan metabolism | 41 | 6 | 9.78E-09 | 8.01 | 7.43E-08 | 0.37 |
| Arginine and proline metabolism | 38 | 4 | 8.27E-11 | 10.10 | 1.12E-09 | 0.34 |
| Nicotinate and nicotinamide metabolism | 15 | 1 | 1.38E-06 | 5.86 | 4.66E-06 | 0.19 |
| Retinol metabolism | 16 | 1 | 1.76E-04 | 3.75 | 2.38E-04 | 0.16 |
| Arginine biosynthesis | 14 | 3 | 4.50E-12 | 11.3 | 1.21E-10 | 0.14 |
| Citrate cycle (TCA cycle) | 20 | 2 | 2.06E-05 | 4.69 | 3.50E-05 | 0.14 |
| Tyrosine metabolism | 42 | 1 | 1.03E-02 | 1.99 | 1.07E-02 | 0.14 |
| Alanine, aspartate and glutamate metabolism | 28 | 2 | 1.10E-08 | 7.96 | 7.43E-08 | 0.11 |
| Cysteine and methionine metabolism | 33 | 1 | 2.08E-05 | 4.68 | 3.50E-05 | 0.10 |

| **Table S2.** Analyzed pathways of metabolomics data differently regulated in serum of MHD mice  compared with model mice. | | | | | | |
| --- | --- | --- | --- | --- | --- | --- |
|  | Total Cmpd | Hits | Raw *p* | -log(p) | FDR | Impact |
| Phenylalanine, tyrosine and tryptophan biosynthesis | 4 | 1 | 8.17E-03 | 2.09 | 8.17E-03 | 0.50 |
| Phenylalanine metabolism | 12 | 1 | 8.17E-03 | 2.09 | 8.17E-03 | 0.36 |
| Arginine and proline metabolism | 38 | 3 | 2.05E-04 | 3.69 | 3.36E-04 | 0.28 |
| Nicotinate and nicotinamide metabolism | 15 | 1 | 9.02E-05 | 4.04 | 2.01E-04 | 0.19 |
| Tryptophan metabolism | 41 | 4 | 2.47E-05 | 4.61 | 9.86E-05 | 0.18 |
| Retinol metabolism | 16 | 1 | 4.86E-05 | 4.31 | 1.40E-04 | 0.16 |
| Citrate cycle (TCA cycle) | 20 | 2 | 9.21E-06 | 5.04 | 4.61E-05 | 0.14 |
| Cysteine and methionine metabolism | 33 | 1 | 2.29E-03 | 2.64 | 2.54E-03 | 0.10 |
